# miR-450b-5p induced by oncogenic KRAS is required for colorectal cancer progression

**DOI:** 10.18632/oncotarget.11016

**Published:** 2016-08-02

**Authors:** Ya-Ping Ye, Ping Wu, Chun-cai Gu, Dan-ling Deng, Hong-Li Jiao, Ting-Ting Li, Shu-Yang Wang, Yong-Xia Wang, Zhi-Yuan Xiao, Wen-ting Wei, Yan-Ru Chen, Jun-Feng Qiu, Run-Wei Yang, Jie Lin, Li Liang, Wen-Ting Liao, Yan-Qing Ding

**Affiliations:** ^1^ Department of Pathology, Nanfang Hospital, Southern Medical University, Guangzhou, Guangdong, China; ^2^ Department of Pathology, School of Basic Medical Sciences, Southern Medical University, Guangzhou, Guangdong, China; ^3^ Guangdong Province Key Laboratory of Molecular Tumor Pathology, Guangzhou, Guangdong, China

**Keywords:** miR-450b-5p, Wnt/β-Catenin pathway, colorectal cancer, progression, KRAS

## Abstract

The development and progression of CRC are regarded as a complicated network and progressive event including genetic and/or epigenetic alterations. Recent researches revealed that MicroRNAs are biomarkers and regulators of CRC progression. Analyses of published microarray datasets revealed that miR-450b-5p was highly up-regulated in CRC tissues. In addition, high expression of miR-450b-5p was significantly associated with KRAS mutation. However, the role of miR-450b-5p in the progression of CRC remains unknown. Here, we sought to validate the expression of miR-450b-5p in CRC tissues and investigate the role and underlying mechanism of miR-450b-5p in the progression of CRC. The results revealed that miR-450b-5p was up-regulated in CRC tissues, high expression level of miR-450b-5p was positively associated with poor differentiation, advanced TNM classification and poor prognosis. Moreover, miR-450b-5p was especially high in KRAS-mutated cell lines and could be up-regulated by KRAS/AP-1 signaling. Functional validation revealed that overexpression of miR-450b-5p promoted cell proliferation and tumor growth while inhibited apoptosis of CRC cells. Furthermore, we demonstrated that miR-450b-5p directly bound the 3′-UTRs of SFRP2 and SIAH1, and activated Wnt/β-Catenin signaling. In conclusion, miR-450b-5p induced by oncogenic KRAS is required for colorectal cancer progression. Collectively, our work helped to understand the precise role of miR-450b-5p in the progression of CRC, and might promote the development of new therapeutic strategies against CRC.

## INTRODUCTION

Colorectal cancer (CRC) is one of the most commonly digestive malignant tumor worldwide and the incidence of which increases rapidly among different kinds of cancer in recent decades [1]. Although survival rates of CRC patients with early stage disease have improved in the last few years, the clinical outcome of CRC patients with advanced stage disease still remains poor [1, 2]. Therefore, there is a pressing need for more effective treatment options.

The development and progression of the most sporadic CRC follow the classical adenoma-carcinoma sequence which is a result of the accumulation of genetic mutations and epigenetic alterations [3]. Mutations on tumor suppressor adenomatous polyposis coli (APC), KRAS, BRAF, or TP53 genes have been characterized as key factors of CRC cancer-initiating [3–5]. Deletion or mutations in APC or stabilizing mutations in β-Catenin lead to intracellular β-Catenin accumulation and constitutively activate the Wnt/β-Catenin signaling [6, 7], which in turn stimulates the expression of a number of target genes that drive tumorigenesis [8, 9].

Activation of the Wnt signaling pathway caused by mutations in these genes has been seen in over 85% of sporadic CRC patients [10]. However, it is interesting that heterogeneous activation of Wnt/b-catenin signaling exists in individual CRC tumors. Nuclear accumulation of b-Catenin, one of the hallmarks of Wnt/β-catenin activation, significantly increased in those dedifferentiated tumor cells at the edge of CRC tissues. In contrast, less intracellular accumulation of β-Catenin was observed in the central areas of tumor masses [11, 12]. This kind of dynamic status of Wnt/β-catenin signaling can be difficultly explained simply by genetic mutations in APC or β-Catenin in CRC. Actually, alternative regulations of Wnt/β-Catenin signaling in CRC cells with mutations in APC or β-Catenin have been identified. For example, Wnt/β-Catenin activity induced by mutant APC or β-Catenin can be partially inhibited by upstream secreted Frizzled-related proteins (SFRPs) [13], and overexpression of Axin can down-regulate β-Catenin in APC-mutated CRC cells [14]. In addition, the ubiquitin proteasome degradation is a main regulatory pathway for β-catenin. SIAH1 binds with the carboxyl end of APC and promotes the degradation of β-catenin [15, 16]. These researches suggest that Wnt/β-catenin signaling can be activated or inhibited at many levels with a wide and dynamic range.

MicroRNAs (miRNAs) are a class of highly conserved single-stranded noncoding RNAs that regulate protein expression at the posttranslational level through binding to the 3′-untranslated region (UTR) of their target mRNAs [17, 18]. Recent researches have revealed that miRNAs are biomarkers and regulators of CRC progression [19]. Additionally, a small group of miRNAs has been proved to be involved in tumorigenesis or progression of CRC through modulation of Wnt/β-Catenin signaling pathways [20–25].

Recently, miR-450b-5p has been shown to be associated with proliferation, differentiation as well as chemo-resistance of some cancer cells [26, 27], and our preliminary work and published microarray analysis also indicated that the expression of miR-450b-5p was up-regulated, and it can induce activation of Wnt/β-Catenin signaling in CRC. But the role of miR-450b-5p in CRC progression and the molecular mechanisms about regulating the activation of Wnt/β-Catenin signaling are unclear.

Herein, our study suggested that miR-450b-5p induced by KRAS took part in the progression of CRC cells with mutations in APC or β-Catenin, and high expression level is associated with aggressive phenotype and poor prognosis of patients with CRC. Further investigations revealed that miR-450b-5p directly bound the 3′-UTRs of SFRP2 and SIAH1, which function as regulators of Wnt/β-Catenin signaling, boosting the activation of Wnt/β-Catenin signaling, and promoting the progression of CRC.

## RESULTS

### Overexpression of miR-450b-5p correlates with CRC progression

**Figure 1:**
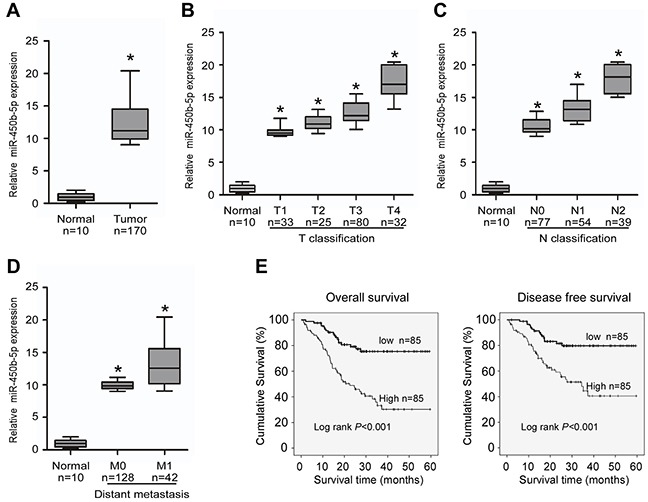
Overexpression of miR-miR-450b-5p correlates with CRC progression Real-time PCR analyses of miR-450b-5p in 10 normal intestine epithelial tissues (normal) and 170 CRC tissues (tumor), normalized by U6 expression. Boundaries of boxes represent bounding of the boxes stand for the lower and upper quartile. Lines within the boxes and whiskers represent median and extremum. **A.** Mean expression of miR-450b-5p in normal tissues (normal) and CRC tissues (tumor). **B.** Expression of miR-450b-5p in different T classification (T1-T4) of CRC compared with 10 normal intestine tissues. **C.** Expression of miR450b-5p in different N classification (N0-N2) of CRC. **D.** Expression of miR-450b-5p in different distant metastasis stage of CRC. **E.** Overall survival and disease-free survival time curves analyzed by Kaplan-Meier of patients with high (≥median; n=85) or low miR-450b-5p (<median; n=86) expression; median= 5.84, p<0.001. * p<0.05.

Several published CRC miRNA microarray datasets showed that miR-450 was among the highest microRNAs in CRC tissues [28]. Analyses of GEO (http://www.ncbi.nlm.nih.gov/geo/) CRC miRNA microarray datasets also revealed that miR-450b-5p was markedly overexpressed in primary CRC tissues as compared with normal colon tissues (Supplementary Figure S1, NCBI/GEO/GSE46622 and GSE28364; n=16 and n=80). We then validated the expression of miR-450b-5p in human CRC tissues samples. In agreement with the analytical results from the published CRC miRNA microarray datasets, our analysis also uncovered that miR-450b-5p was dramatically overexpressed in 170 primary CRC tissues compared with 10 normal colon tissues (Figure 1A).

Next, we explore the clinicopathological significance of miR-450b-5p. The results indicated that high expression of miR-450b-5p was positively associated with advanced TNM stage in patients with CRC (Figure 1B-1D, Supplementary Table S1 and S2). According to the median ratio (5.84) of relative miR-450b-5p expression, the 170 samples were classified into two groups: the high-miR-450b-5p group (n=85, miR-450b-5p expression ratio> median ratio), and the low-miR-450b-5p group (n=85, miR-450b-5p expression ratio<mean ratio). The survival analysis indicated that the group of high miR-450b-5p expression had shorter 5-year overall survival and 5-year disease-free survival (Figure 1E, log-rank, P<0.001), and the benefit for survival from low miR-450b-5p expression is an independent effect for patients with CRC (Supplementary Table S3).

### KRAS signaling enhances miR-450b-5p expression in CRC

**Figure 2:**
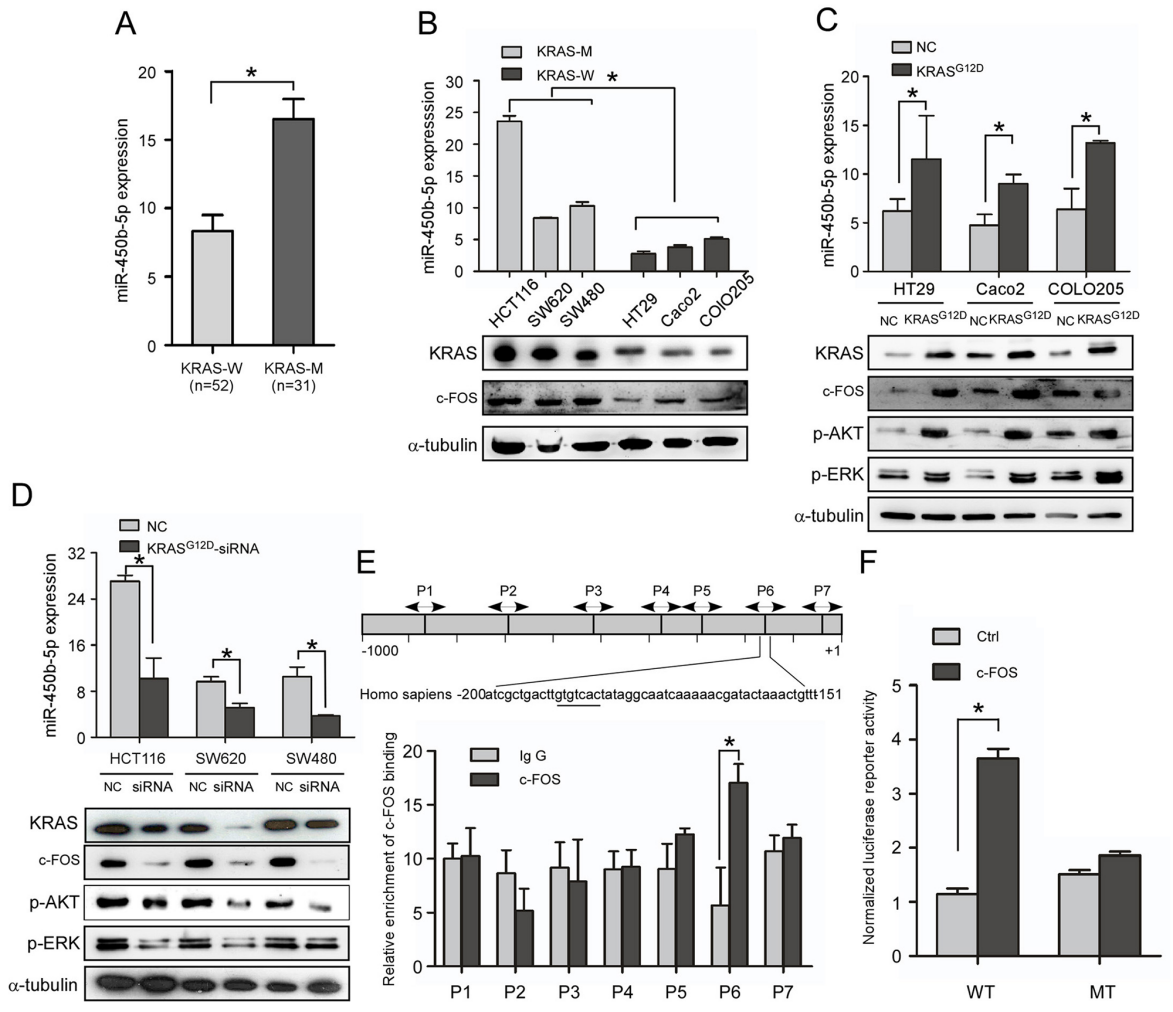
KRAS signaling enhances miR-450b-5p expression in CRC **A.** Real-time PCR analyses of miR-450b-5p expression in CRC samples with wild-type KRAS (n=52) and CRC samples with mutant-KRAS (n=31). **B.** Real-time PCR analyses of miR-450b-5p expression and western blotting analyses of KRAS expression in indicated CRC cell lines with different KRAS types. **C, D.** Real-time PCR analyses of miR-450b-5p expression and western blotting analyses of KRAS and its downstream genes expression in indicated cells transfected with mutant KRAS^G12D^ or treated with KRAS^G12D^-siRNA. **E.** Chromatin immunoprecipitation assay (CHIP) for detection of c-FOS binding site on the promoter of miR-450b-5p. **F.** Luciferase activity analyses of c-FOS on promoter activity of miR-450b-5p transfected with wild-type and mutated-type reporter vector.

When the expression of miR-450b-5p in CRC was analyzed using published miRNA microarray datasets (NCBI/GEO/GSE28364; n=80), an interesting result was found that the miR-450b-5p was significantly higher in KRAS mutant CRC samples than that in KRAS wild CRC samples (Supplementary Figure S2, P<001, n=40), which was proved by Q-PCR in CRC tissue samples (Figure 2A, P<001, n=83). Moreover, we detected the expression of miR-450b-5p in KRAS wild and KRAS mutant CRC cell lines, the results suggested that the expression of miR-450b-5p in CRC cell lines with KRAS mutation (HCT116, SW2620 and SW480) was much higher than that in cell lines with wild type KRAS (HT29, Caco2 and COLO250) (Figure 2B, P<0.01), and there was no significant correlation between the expression level of miR-450b-5p and the type of KRAS mutation (Supplementary Figure S3). Meanwhile, the expression of miR-450b-5p was obviously increased in the KRAS wild CRC cells with mutant KRAS^G12D^ transduction (Figure 2C, P<0.01), whereas it was decreased in the KRAS mutant CRC cells treated with KRAS^G12D^-siRNA (Figure 2D, P<0.01). These results indicated that miR-450b-5p might be up-regulated upon KRAS mutation in CRC cells.

We next investigated the potential molecular mechanism through which KRAS may up-regulate miR-450b-5p. Bioinformatics' analysis revealed a putative c-FOS binding site in the promoter of miR-450b-5p gene at −151 to −200 bp. C-FOS is key member of the transcription factor AP-1, which is a well-known downstream gene of KRAS signaling. We then tested whether c-FOS could bind with the promoter of miR-450b-5p by ChIP assay using seven pairs of primers covering −1000 to +1 bp of miR-450b-5p promoter. The results indicated a putative 50 bp fragment in miR-450b-5p promoter (−200 to −151) as a potential binding region of miR-450b-5p, which contains the predictive binding site (Figure 2E). Further luciferase reporter assay showed that the predictive site −200bp to −151bp in miR-450b-5p promoter bound with AP-1, whereas the mutants of bp −200 to −150 showed no binding with AP-1 (Figure 2F). Moreover, the expression of miR-450b-5p was coordinated with c-FOS (Figure 2B-2D, Supplementary Figure S4). These results indicate that AP-1 could up-regulate miR-450b-5p through direct binding with the promoter of miR-450b-5p.

### miR-450b-5p promotes CRC progression in vitro and in vivo

**Figure 3:**
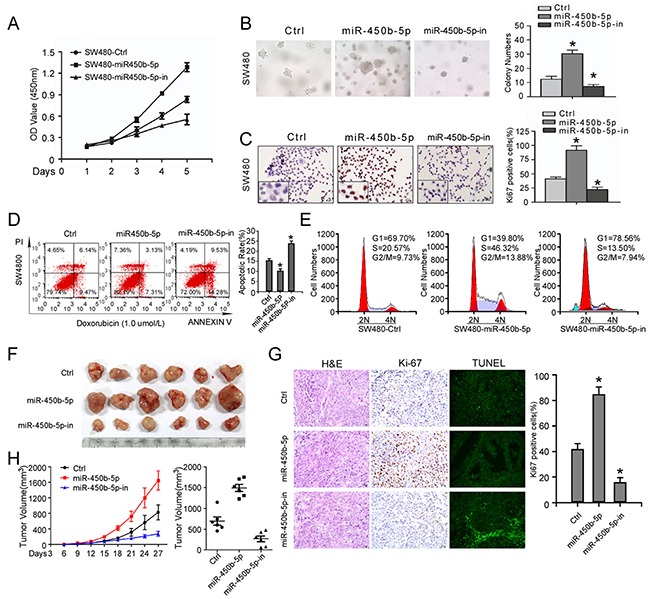
miR-450b-5p promotes CRC aggressiveness in vitro and in vivo **A.** MTT assays performed on indicated cells. **B.** Anchorage-independent colony-formation assays performed on indicated cells. The number of colonies>0.1 mm in diameter was scored. **C.** Transwell assay performed on indicated cells and the quantification of Ki-67 positive cells by IHC staining. **D.** Flow-cytometry of apoptosis assay on cells treated with 1.0μM doxorubicin (left panel) and Annexin-positive/PI-negative cells were calculated for apoptotic rate (right panel). **E.** Cell cycle assay performed by flow-cytometry of the indicated CRC cells treated with 0.1μM colchicine. **F.** Images of tumor from nude mice injected with SW480. **H.** Tumor volume of nude mice measured every three days (left panel) and final tumor volume in each group (right panel). Data points are presented as the mean tumor volume ± SD. **G.** left panel: **H&E** staining, IHC staining with antibody against Ki-67 and TUNEL staining of sections from xenograft tumors respectively; right panel: the proliferation index (PI) of the indicated cancer cells (quantification of Ki-67-positive cells). Error bars represent mean ± SD of three independent experiments. * p<0.05.

We explore the function of miR-450b-5p over-expression in CRC progression. Over-expression of miR-450b-5p obviously accelerated, whereas inhibition of miR-450b-5p decreased, growth rate, anchorage-independent growth and proliferation index (calculated by Ki-67 expression) ability of CRC cells (Figure 3A-3C, Supplementary Figure S5A-S5C). Meanwhile, the stimulatory effect of miR-450b-5p on apoptosis was analyzed. The results showed that overexpression of miR-450b-5p obviously reduced, whereas inhibition of miR-450b-5p increased, apoptosis rate of CRC cells (Figure 3D-3E, Supplementary Figure S5D-5E and Supplementary Figure S6).

We also engineered SW480 cells to stably express miR-450b-5p and miR-450b-5p-inhibitor, and then the engineered CRC cells were injected into the dorsal flank of nude mice. The results showed the tumors from miR-450b-5p-overexpressing SW480 cells were much larger, whereas formed by miR-450b-5p-inhibited cells were smaller. Meanwhile, miR-450b-5p-overexpressing tumors showed an increased proliferation index and decreased apoptotic rate (Figure 3F-3H, Supplementary Figure S5F-5H), whereas miR-450b-5p-inhibited tumors displayed a lower proliferation index and a higher apoptotic rate (Figure 3G and Supplementary Figure S5G). These results demonstrate that miR-450b-5p overexpression promotes CRC tumor growth in vivo.

### miR-450b-5p activates Wnt signaling pathway in CRC

**Figure 4:**
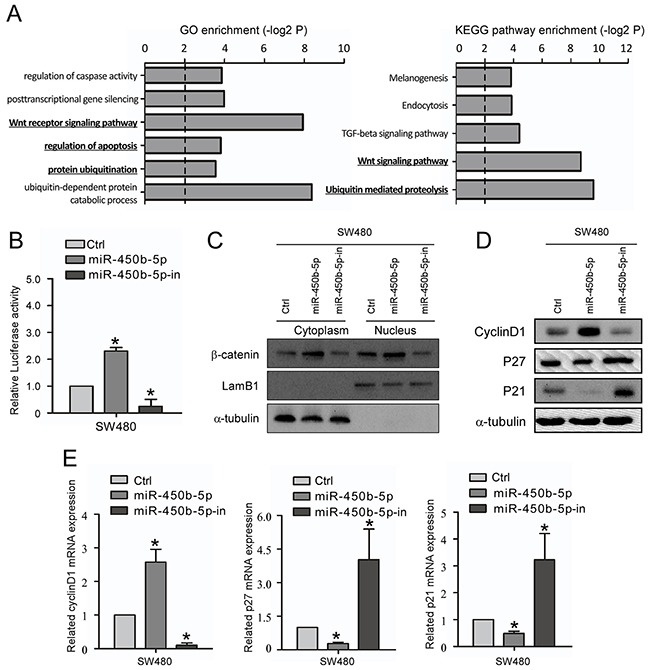
miR-450b-5p activates Wnt signaling pathway in CRC **A.** GO enrichment and KEGG enrichment of pathways involving predicted miR-450b-5p targeting genes. **B.** The Wnt signaling luciferase reporter assay of indicated cells transfected with miR-450b-5p and miR-450b-5p-inhibitor. **C.** Western blotting assay for β-Catenin in cytoplasm and nucleus of indicated cells transfected with control, miR-450b-5p and miR-450b-5p-inhibitor. LamB1 and a-tubulin served as loading controls for nucleus and cytoplasm proteins, respectively. **D.** Western blot analysis in indicated cells of protein products of Wnt signaling pathway downstream genes. **E.** Q-PCR analysis in indicated cells of protein products of Wnt signaling pathway downstream genes.* p<0.05.

Bioinformatics analysis of these genes revealed many biological processes and pathways that were potentially involved in the progression of CRC, including the GO terms proteolysis, apoptosis, notably the KEGG pathways Wnt signaling pathway and ubiquitin mediated proteolysis (Figure 4A).

Next, the effect of miR-450b-5p on location of β-catenin and activity of Wnt/β-Catenin signaling were analyzed. The results of the Wnt signaling luciferase reporter assay showed that ectopic expression of miR-450b-5p remarkably increased the luciferase activity, while inhibition of miR-450b-5p repressed the luciferase activity (Figure 4B). In addition, miR-450b-5p increased the β-catenin expression level both in cytoplasm and in nuclear (Figure 4C, Supplementary Figure S7). Moreover, the expression of Wnt signaling downstream genes was also analyzed. The results showed that ectopically expressing of miR-450b-5p significantly increased the expression of Cyclin D1 but decreased the expression of P21 and P27; instead, overexpression of miR-450b-5p-inhibitor remarkably decreased the expression of Cyclin D1 but increased the expression of P21 and P27 (Figure 4D, 4E).

### miR-450b-5p directly binds the 3′-UTRs of SIAH1 and SFRP2 and inhibits their expression

**Figure 5:**
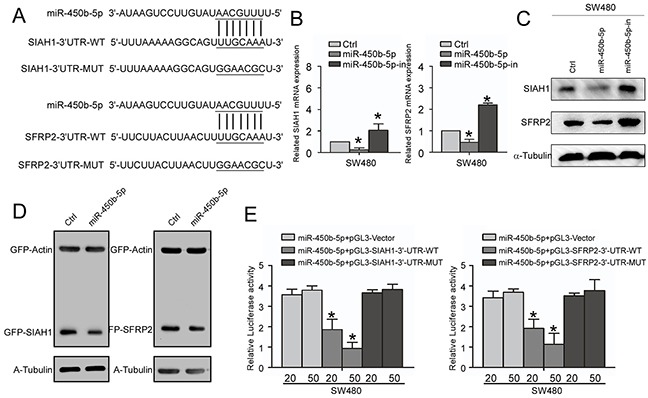
miR-450b-5p directly targets SIAH1 and SFRP2 **A.** Predicted miR-450b-5p target sequences in the 3′-UTR of SIAH1 (SIAH1-3′UTR-WT) and SFRP2 (SFRP2-3′UTR-WT), and sequences with mutated nucleotides (SIAH1-3′UTR-MUT and SFRP2-3′UTR-MUT). **B.** Real-time PCR analysis of SIAH1 and SFRP2 expression, normalized by GAPDH. **C.** Western blotting analysis of SIAH1 and SFRP1 in indicated cells. **D.** Western blotting analyses of GFP proteins in indicated cells. α-Tubulin served as loading control. **E.** Luciferase activity analysis of indicated cells transfused with indicated reporters of a different amount of miR-450b-5p (20 and 50 nM). Data were presented in mean ± SD of three independent experiments. * p<0.05.

On analyzing the predicted targets using public algorithms (TargetScan and miRANDA), we noticed that SIAH1 and SFRP2 might be key targets of miR-450b-5p that might involve in regulation of Wnt signaling (Figure 5A). We then validated the effect of miR-450b-5p on SIAH1and SFRP2 expression. The results showed that miR-450b-5p decreased the expression, whereas miR-450b-5p inhibitor increased the expression of SIAH1and SFRP2 at both transcriptional and translational levels (Figure 5B and 5C). When tested using the dual luciferase reporter vectors containing a complete wild-type SIAH1-3′-UTR and SFRP2-3′-UTR, miR-450b-5p inhibited the expression of GFP, but not the control in CRC SW480 cells (Figure 5D). Furthermore, the luciferase reporter assay also demonstrate that over-expression of miR-450b-5p dramatically inhibited the luciferase activity of the wild SIAH1-3′-UTR and SFRP2-3′UTR in a dose-dependent manner, and ectopic expression of miR-450b-5p had no the same effect on the mutant SIAH1-3′-UTR and SFRP2-3′ UTR (Figure 5E).

### Repression of SIAH1 and SFRP2 inhibits the CRC progression induced by miR-450b-5p

**Figure 6:**
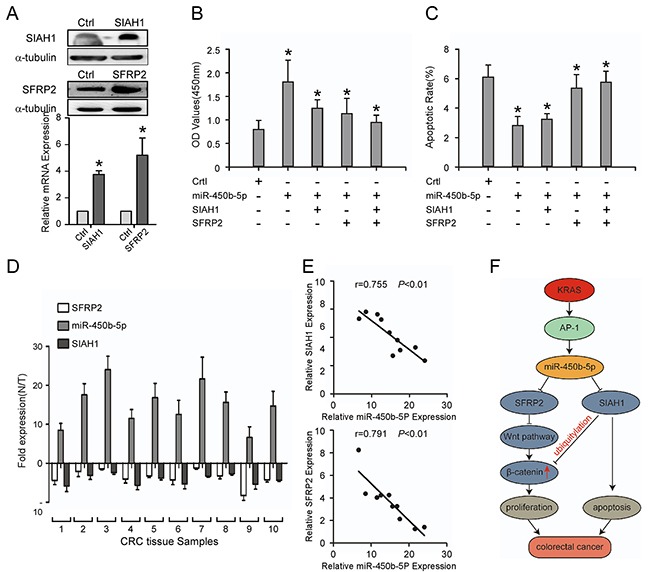
Repression of SIAH1 and SFRP2 inhibits the CRC progression induced by miR-450b-5p, and the clinical relevance of miR-450b-5p and its targets in CRC **A.** Western blotting and Real-time PCR analyses of SIAH1 and SFRP2 exogenous expression. **B.** MTT assays on indicated cells. The OD values (450 nm) of cells at day 7 were analyzed; **C.** Flow-cytometry of an apoptosis assay on indicated cells. Annexin-positive/PI-negative cells were calculated for apoptotic rate. **D.** Real-time PCR analyses of SFRP2, miR-450b-5p, and SIAH1 expression in 10 fresh human CRC samples. **E.** Spearman correlation analyses on relative expression of miR-450b-5p and relative expression of SIAH1 and SFRP2 in 10 fresh human CRC samples. **F.** Proposed model: miR450b-5p is increased by mutated KRAS through AP-1 binding to its promoter, and then down-regulates SIAH1 and SFRP2, finally activating Wnt signaling pathway. Error bars represent mean ± SD from three independent experiments, * p<0.05.

To further confirm the role of miR-450b-5p in the progression of CRC, SIAH1 and SFRP2 ORF constructs without 3′UTRs were transfected in SW480 cells with miR-450b-5p overexpressing (Figure 6A), and their effects on growth and apoptotic rate were detected. The restoration experiment indicated that re-expression of SIAH1 or/and SFRP2 decreased the growth rate of SW480 cell (Figure 6B). However, the re-expression of SIAH1 or/and SFRP2 increased apoptotic rate of SW480 cell (Figure 6C).

Then the correlation between the expression of miR-450b-5p and the expression of SIAH1 and SFRP2 was analyzed in 10 freshly CRC tissues. The results indicated that the expression of miR-450b-5p negatively correlated with the expressions of SIAH1 (Figure 6D, r=−0.755, P<0.001) and SFRP2 (Figure 6E, r=−0.791, P<0.001).

Taken altogether, the above results revealed that AP-1, a downstream gene of KRAS signaling, up-regulates the expression of miR-450b-5p, which activates Wnt signaling and inhibits apoptosis by decreasing the expression of SIAH1 and SFRP2, resulting in CRC progression (Figure 6G).

## DISCUSSION

Activation of the KRAS and Wnt signaling pathways are two key genetic events underlying the development of CRC. The two pathways can cooperate to promote the progression of CRC. Some studies have previously shown that oncogenic KRAS signaling promotes the Wnt/β-catenin pathway through LRP6 and GSK-3β in CRC [29, 30]. Moreover, it is well known that microRNAs inhibit genes expression and pathways crosstalk, and increasing researches indicate that microRNAs participate in the progression of many kinds of human malignant cancers [31, 32]. However, the function of microRNAs for the KRAS and Wnt signaling pathways interaction in CRC still remains puzzling. Herein, we demonstrate that miR-450b-5p up-regulated by KRAS activate Wnt/β-Catenin signaling in CRC cells.

So far, there has been little discussion about the function of miR-450b in pathogenesis of cancer. Recent studies have shown that miR-450b functions as a new tumor repressor, suppressing the proliferation of tumor cells in breast cancer and rhabdomyosarcoma [26, 33], and miR-450b-5p inhibits stemness and the development of chemoresistance in CRC [27, 34]. However, our results and published microarray analysis [35, 36] showed that the miR-450b-5p expression was up-regulated in CRC, and was associated with aggressive phenotype and poor patient prognosis. Moreover, ove-rexpression of miR-450b-5p significantly promoted, whereas inhibition of miR-450b-5p impaired, CRC cell growth rate in vitro and vivo. Therefore, these results show that miR-450b-5p may serve as an oncogene in CRC progression. These opposite effect of miR-450b-5p on tumorigenesis in different cancers may lie in the different cell types and its target genes (different genes activated in different stages of the cancer progression) [37] [38].

Over the past decade, Wnt/β-Catenin signaling is considered as a hallmark of colorectal tumorigenesis [39–41]. Our results show that miR-450b-5p activates Wnt/β-Catenin signaling by directly binding the 3′-UTRs of SIAH1. SIAH1 has emerged as a tumor suppressor, inhibits cell proliferation and promotes apoptosis, by binding with APC and promoting the degradation of β-catenin [16, 42]. Moreover, our results also suggest that miR-450b-5p activates Wnt/β-Catenin signaling by decreasing the expression SFRP2, another negative modulator of Wnt pathway, and its expression can be inhibited by methylation and microRNAs in CRC and other malignant cancers [43–45]. We restored the expression of SIAH1 and SFRP2 in miR-450b-5p-overexpressing CRC cells, which showed that overexpression of SIAH1 and SFRP2 remarkably reduced the cells growth rate, and promoted apoptosis. Taken together, miR-450b-5p promotes CRC progression through activation of Wnt/β-Catenin signaling by decreasing the expression of SIAH1 and SFRP2.

MiR-450b-5p expression was up-regulated in CRC; however, how miR-450b-5p was up-regulated in tumor cells still remains unknown. Analyzing the expression of miR-450b-5p in CRC, we also noticed that the miR-450b-5p was dramatically higher in KRAS wild CRC samples than that in KRAS mutant CRC samples. Therefore, we speculated that KRAS activating mutations may increase the expression of miR-450b-5p in CRC. KRAS is an important and frequently mutated gene during CRC progression [46, 47], and its activating mutations result in CRC cells proliferation, invasion and metastasis [48–50].

The transcription factor AP-1 is a downstream gene of KRAS signaling. Some reports have implicated that c-FOS (a member of the transcription factor AP-1 component) plays an important role in the tumorigenesis of several human cancers. For example, it has been reported that knockdown of c-FOS suppresses the growth of CRC cells in nude mice [51], and c-FOS interacting with other proteins enhances the transcription of IL-6 and VEGF-A, and promotes angiogenesis in CRC [52]. Our present study showed that miR-450b-5p in CRC cells was up-regulated by direct binding of c-FOS to the promoter region of miR-450b-5p gene to initiate its transcription. This reveals a mechanistic basis for miR-450b-5p overexpression in CRC cells. Thus, disruption of KRAS/AP-1-induced miR-450b-5p expression may contribute to the development of anti-CRC therapy.

In conclusion, we demonstrate that miR-450b-5p induced by oncogenic KRAS is required for colorectal cancer progression. Understanding the precise function of miR-450b-5p in the development of CRC and the specific molecular mechanism of the Wnt/β-Catenin pathway activation will enriching our knowledge about CRC progression, and may provide theoretical evidence for individualized treatment and development of molecule-targeting agents of CRC.

## MATERIALS AND METHODS

### Clinical specimen

The clinical research of samples was performed according to the written approval obtained from the Southern Medical University Institutional Board (Guangzhou, China). All the specimens were collected with informed consent of patients. CRC tissue samples (n=170) were collected between 2008 and 2010, and 50 CRC tissues and the matched adjacent normal tissues were obtained between 2014 and 2015 at the Department of Pathology, Southern Medical University. Surgically resected tissues were frozen in liquid nitrogen immediately until future analysis. The medical records of patients were reviewed for acquisition of the clinicopathological information: age, gender, differentiation, and TNM stage. Survival data were available for the cohort of 170 patients. The median follow-up time was 23 (range, 1-59.6) months.

### Cell culture

Human CRC cell lines were purchased from The Global Bioresource Center (ATCC, USA). COLO205 was cultured in RPMI 1640 medium (Gibco, Grand Island, NY, USA) containing 10% fetal bovine serum (Gibico, Grand Island, NY, USA); SW620 and SW480 cells were cultured in Leibovitz's L-15 Medium supplemented with 10% FBS (Gibico); HT29, HCT116 cells were cultured in McCoy's 5a Medium Modified with 10% FBS (Gibico); Caco-2 cell was cultured in Dulbecco's modified Eagle's medium (DMEM; Gibco) with 10% FBS (Gibico). All the cells were cultured at 37°C, 5% CO_2_.

### Real-time PCR

The total miRNA was extracted from tissues or cells using mirVana miRNA Isolation Kit (Ambion, Austin, TX, USA) following the manufacturer's instructions. After that, cDNA was generated from total miRNA using the Tapman miRNA Reverse Transcription Kit (Applied Biosystems, Foster City, CA, USA). Real-time quantitative PCR was performed using the Applied Biosystems 7500 sequence detection system iQTM SYBR Green Supermix (BioRad Laboratories, Hercules, CA, USA) containing 5ng cDNA and 10pM of each primer, under the cycling conditions: 5min at 94°C, 40 cycles (30s at 95°C, 30s at 56°C). The expression of mir-450b was measured using Ct (threshold cycling) value and its relative quantification was calculated by normalizing with U6 (internal control of small nuclear RNA expression) as 2^−[(CT of mir-450b-5p)-(CT of U6)]^. For quantification of other genes, RT-qPCR was performed as described preciously [31]. The primers used are listed in Supplementary Table S4.

### Western blotting

Proteins were isolated, subjected to SDS-page, transferred onto PVDF membranes and incubated with antibodies anti-SIAH1 (Abcam, Cambridge, MA, USA), anti-SFRP2 (Cell Signaling Technology, Danvers, MA, USA), anti-KRAS (Proteintech, USA), anti-Ki-67 (Abcam, Cambridge, MA, USA), anti-p27, anti-p21, anti-cyclinD1 (Bioworld Technology Inc. St. Louis Park, MN, USA). LamB1 and a-tubulin (Sigma, Saint Louis, MO, USA) were used as loading controls. Then, the membranes were detected by chemiluminescence. All operations above were performed in accordance with standard methods.

### Plasmids and transfection

To construct the plasmid that overexpresses mir-450b-5p, a 78bp fragment of pri-mir-450b-5p was PCR-amplified, then generated into the lentiviral vector pLVTHM (Addgene Inc., Cambridge, MA, USA). The mimics, negative control and inhibitor of mir-450b-5p were purchased from Genecopoeia (Guangzhou, Guangdong, China), and transfected into cells with Lipofectamine 2000 reagent (Invitrogene), according to the manufacturer's instruction. For luciferase assay, small regions containing the target sequences of mir-450b-5p in target genes 3′-UTR were generated by PCR amplification and cloned into pGL3-basic luciferase reporter plasmid (Promega). Two concentrations of mir-450b-5p mimics or inhibitors (20 and 50 nM) were applied. The primers are listed in Supplementary Table S5.

### MTT assay, colony formation assay, soft-agar colony formation assay, flow cytometry assay, luciferase assay

The mimics, inhibitor, and negative control oligos of mir-450b-5p were transfected into cells for MTT assay, colony formation assay, soft-agar colony formation assay, flow cytometry assay and luciferase assay. For details, please see supplementary materials.

### Terminal transferase dUTP nick end labeling (TUNEL) assay

The TUNEL assay was used to detect DNA degradation of nuclear chromatin in apoptotic cells, according to the manufacturer's instructions (Promega, USA). In brief, 5×10^4^ cells were seeded on glass coverslips in 24-well plates, cultured for 24 h. Then these cells were treated with doxorubicin (1.0μM) for 12 h and mir-450b-5p mimics or inhibitor oligos were transiently transfected into these cells. After transfection for 24 h, these cells were fixed by paraffin for 1 h, followed by 0.1 % Triton X-100 in phosphate buffered saline (PBS) for 15 min at room temperature (RT). After washing with PBS for 3 min, the slides were incubated with 20μg/ml proteinase K for 20 min at room temperature. The slides were then washed with 1 × PBS and incubated with 1×TdT buffer for approximately 30 min at room temperature. Subsequently, the slides were incubated with 57 μl of mix buffer and 3μl of TdT enzyme for 60 min at 37°C. After being washed three times with PBS, flow cytometry was used to analyze the prepared cells.

### Chromatin immunoprecipitation (CHIP) assay

According to the manufacturer's instructions (Thermo Fisher, USA), cells were seeded in plates for 24 h, and formaldehyde was added drop-wise to the dishes to a final concentration of 0.75% for cross-linking the protein and DNA at RT for 10 min. Glycine was added to a final concentration of 125 mM and the dishes were shaken at RT for 5 min. The cells were then harvested in cold PBS. After centrifugation for 5 min, the supernatant was discarded and the cell pellets were resuspended in the lysis buffer. After sonicating, lysate was centrifuged and the supernatant was immunoprecipitated using a mouse anti-AP-1 antibody (Abcam, Cambridge, MA, USA) or a rabbit polyclonal antibody (Cell Signaling Technology, Danvers, MA, USA) overnight at 4°C. Seven pairs of primers used for qPCR analysis were listed in Supplementary table S6.

### Xenograft model in nude mice

For tumorigenesis assays, stable cell lines were established by a lentiviral-based system (pLVTHM) including miR-450b-5p, miR-450b-5p-inhibitor and negative control. To generate xenograft tumors, the above-mentioned cells (2×10^6^ for each cell line) were subcutaneous injected on the hind limbs of 4-6-week-old female Balb/C athymic mice(nu/nu; Animal Center of Southern Medical University, Guangzhou, China; n=6 for each group). All the mice were housed and maintained under specific pathogen-free conditions, and all operations in experiments were in conformity with institutional guidelines and approved by the Use Committee for Animal Care. Tumor size was measured by a slide caliper, and tumor volume was determined by the formula: 0.44×A×B^2^ (A for diameter of the base of tumor, and B for corresponding perpendicular value). Tumors removed from mice were fixed in formalin (neutral buffered 10%), embedded in paraffin, and prepared into 4μm sections for staining with haematoxylin.

### Statistical analysis

Data were analyzed using SPSS 19.0 for Windows. The Mann-Whitney U-test and Spearman's correlation analyses were applied for analyzing the relationship between the expression of mir-450b-5p and clinicopathological features of CRC cases. The two-tailed paired Student's t-test was used for comparing two experiment groups. 5-year-overall-survival and disease-free survival curves were plotted by the Kaplan-Meier method and compared with the log-rank test. COX's proportional hazard regression model was established for multivariate analysis of the combinational contribution of mir-450b-5p and clinicopathological features to the survival of patients. P<0.05 was considered significant.

### Accession numbers for data sets

The different expression of mir-450b-5p generated in the study came from the GEO database (GSE46622 and GSE28364), The expression of mir-450b-5p in different KRAS genotype reanalyzed in the study came from the GEO database (GSE28364).

## SUPPLEMENTARY MATERIALS METHODS, FIGURES AND TABLES


