# Supplementary material for: miR-450b-5p induced by oncogenic KRAS is required for colorectal cancer progression
Source: Oncotarget. 2016 Aug 2;7(38):61312–24. doi: 10.18632/oncotarget.11016 (PMC5308653; doi:10.18632/oncotarget.11016)
Supplement: Supplementary file 1 [file oncotarget-07-61312-s001.pdf]

miR-450b-5p induced by oncogenic KRAS is required for colorectal cancer progression

SUPPLEMENTARY MATERIALS AND METHODS

RNA isolation, reverse transcription (RT), and real-time PCR

Total RNA was extracted from human tissues and cell lines with the Trizol reagent (Invitrogen, Carlsbad, CA) in accordance with the manufacturer's instruction. Reverse transcription was performed using the SuperScript First-Strand Synthesis System for RT-PCR (Invitrogen, Carlsbad, CA) . Real-time PCR was carried out using SYBR Green I (Applied BioSystems, Foster, CA). Primers used in Real-time PCR were designed by Primer 5. And datas were caculated with 2^{-DDCT} method after normalized to the housekeeping gene GAPDH.

MTT (3-(4,5 -Dimethyl-2-thiazolyl)-2,5 -diphenyl-2H-tetrazolium bromide) assays

1×10^3 cells were seeded on 96-well plates and cultured for 24 hours. 20ul of 5g/L 3-(4,5-dimethylthiazol-z-yl)-2,5-diphenyltetrazolium bromide (MTT, Sigma, St Louis, MO, USA) was added to each well and incubated for 4 hours. After MTT removed, 150ul dimethyl sulphoxide (DMSO, sigma, St, Louis, MO, USA) was added to the wells. The Absorbance was measured at 570 nm with a Microplate Autoreader (Bio-Rad, Hercules, CA , USA). The experiment was repeated three times.

Colony formation assays

Cells were trypsinized and plated on 6-well plates (500 cells/well) and cultured for 2 weeks. The colonies were stained with 1% crystal violet for 30s after fixation with 4% paraformaldehyde for 5 minutes. The number of colonies, defined as >50 cells/colony were counted. Three independent experiments were performed. The data was calculated using paired t test.

Soft agar assay

500 cells were suspended in 2ml complete medium containing 0.3% agar (Sigma, St Louis, MO). Then plated the agar-cell mixture on top of a bottom layer with 1% complete medium agar mixture 10 days later, measured the colonies with an ocular micrometer. Colonies that larger than 0.1mm in diameter were counted. The experiment was repeated independently for three times for each cell line.

Flow-cytometry analysis

Flow-cytometry assay was performed to asses the cell cycle and apoptosis. Cells were seeded into six-well plates (4×10^5cells/well). For cell cycle, in order to synchronize cells into the G2/M phase of the cell cycle, cells were treated with 0.1 µm colchicine for 12 hour, then cells were transfected with mir-450b mimics and inhibitor. After 24 hour, cells were harvested and fixed with 70% cold ethanol. Next ,cells were added bovine pancreatic RNAase to remove total RNA , incubated for 30min at 37°C, added 20 µg/ml propidium iodide (Sigma-Aldrich) and incubated at room temperature for 20min. At last, flow cytometry was used to asses the cell cycle for prepared cells. For apoptosis assay, after cells were seeded into six-well plates, we added doxorubicin (1.0 µm) for 12 h, and trasfected mir-450b/mimics and inhibitor into cells for 24 h. Then cells were treated with the Annexin V-FITC/propidium iodide (PI) Apoptosis Detection Kit (Promega, USA) complying with the manufacturer's instructions and analysed by flow cytometry. All experiments were performed 3 times. Data were averaged for statistical analysis.

Luciferase assay

Cells were seeded into 24-well plates (1×10^5cells/well) and maintained at 37°C for 24 hour. Plasmids were cotransfected into cells using Lipofectamine 2000 reagent (Invitrogen) as pRL-TK Renilla plasmid (Promega) for control plasmid. After 48 hour, Luciferase and Renilla activities were detected by Dual Luciferase Reporter Assay Kit (Promega), according to the manufacturer's instructions. Experiments for each sample were performed 3 times and data were shown as mean±SD.

SUPPLEMENTARY FIGURES AND TABLES

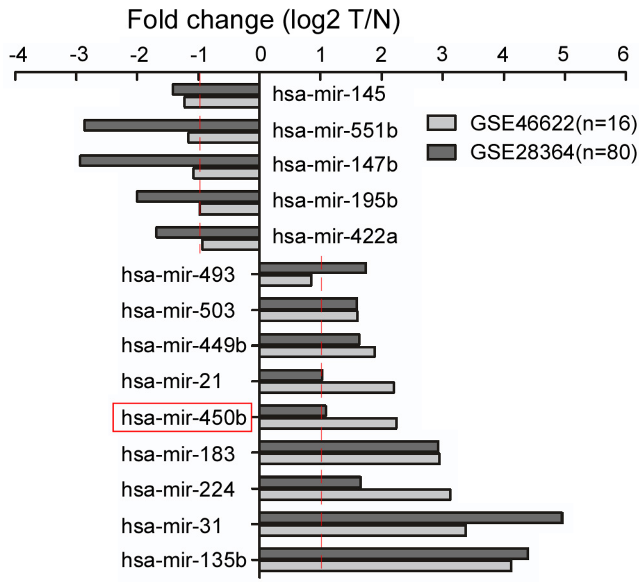

Supplementary Figure S1: Analysis of miR-450b-5p expression in CRC datas from GEO. Analysis of miRNAs expressions were analyzed in Gene Expression Omnibus (GEO, http://www.ncbi.nlm.nih.gov/geo/) data (GSE46622, n=16; GSE28364, n=80) , the result indicated miR-450b-5p was significantly upregulated in CRC samples. Fold change was shown as log2(T/N).

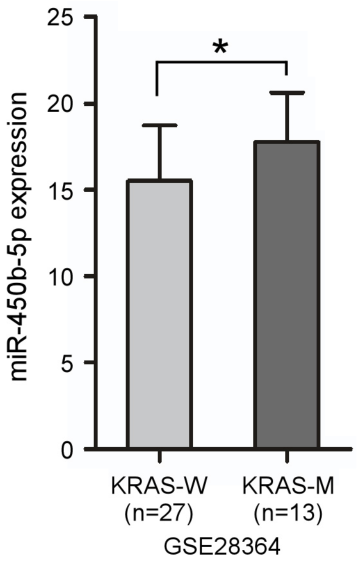

Supplementary Figure S2: miR-450b-5p expression is upregulated in KRAS mutant type CRC. Analysis of miR-450b-5p expression in wild-type KRAS and mutated KRAS in CRC using Gene Expression Omnibus (GEO, http://www.ncbi.nlm.nih.gov/geo/) data (GSE28364, n=40). The result showed that miR-450b-5p expression in CRC samples with wild-type KRAS (n=27) was higer than that with mutated KRAS (n=13). * $p<0.05$.

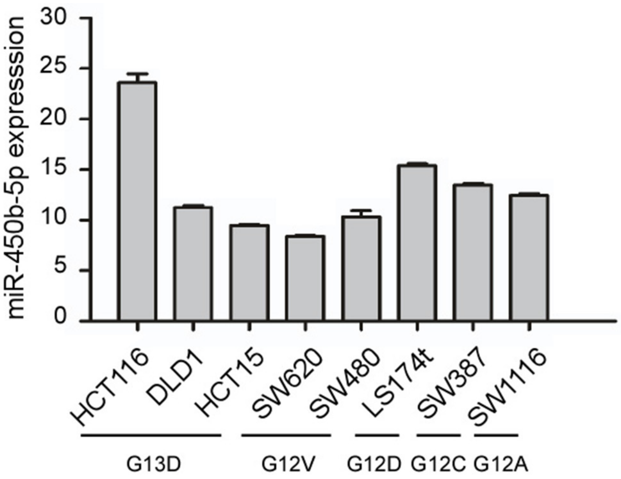

Supplementary Figure S3: The expression level of miR-450b-5p in 8 CRC cell lines with different type of KRAS mutation. Real-time PCR analyses of miR-450b-5p expression in 8 CRC cell lines with different type of KRAS mutation. The result showed that there was no significant correlation between the expression level of miR-450b-5p and the type of KRAS mutation.

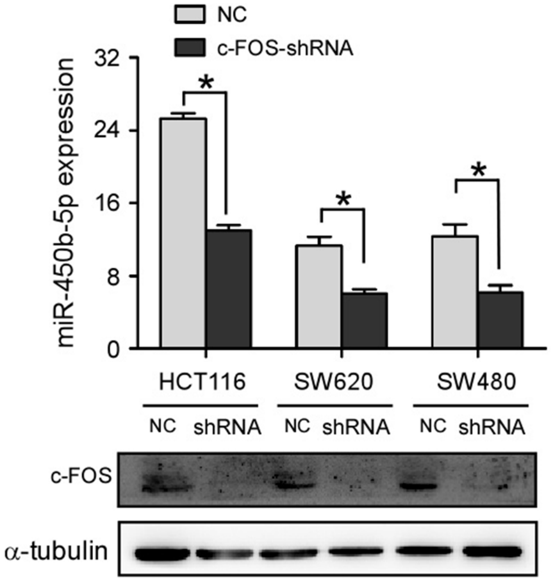

Supplementary Figure S4: c-FOS knockdown decreased the expression of miR-450b-5p in KRAS mutant CRC cell lines. Real-time PCR analyses of miR-450b-5p expression and western blot analyses on c-FOS expression in indicated cells transfected with c-FOS -shRNA.

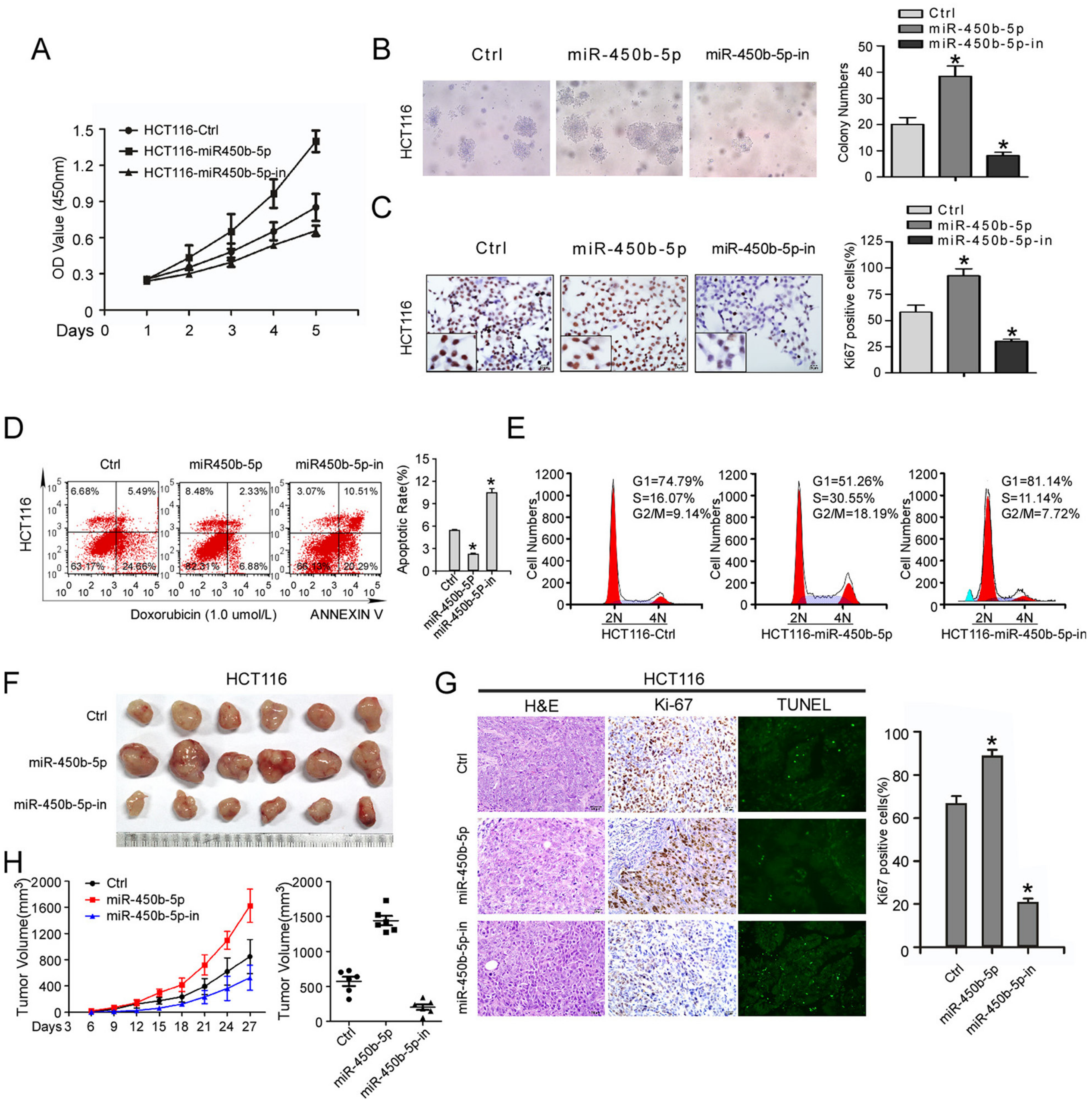

Supplementary Figure S5: miR-450b-5p promotes CRC aggressiveness in vitro and in vivo. A. MTT assays performed on indicated cells. **B.** Anchorage-independent colony-formation assays performed on indicated cells, the number of colonies>0.1 mm in diameter was scored. **C.** Transwell assay performed on indicated cells and the quantification of Ki-67 positive cells by IHC staining. **D.** Flow-cytometry of apoptosis assay on cells treated with 1.0μM doxorubicin (left panel) and Annexin-positive/PI-negative cells were calculated for apoptotic rate (right panel). **E.** Cell cycle assay performed by of the indicated CRC cells treated with 1.0μM doxorubicin. **F.** Images of tumor from nude mice injected with HCT116. **H.** Tumor volume of nude mice measured every three days (left panel) and final tumor volume in each group (right panel), Data points are presented as the mean tumour volume ± SD. **G.** left panel: H&E staining, IHC staining with antibody against Ki-67 and TUNEL staining of sections from xenograft tumors respectively; right panel: the proliferation index (PI) of the indicated cancer cells (quantification of Ki-67-positive cells). Error bar represents mean±SD of three independent experiments. * $p<0.05$.

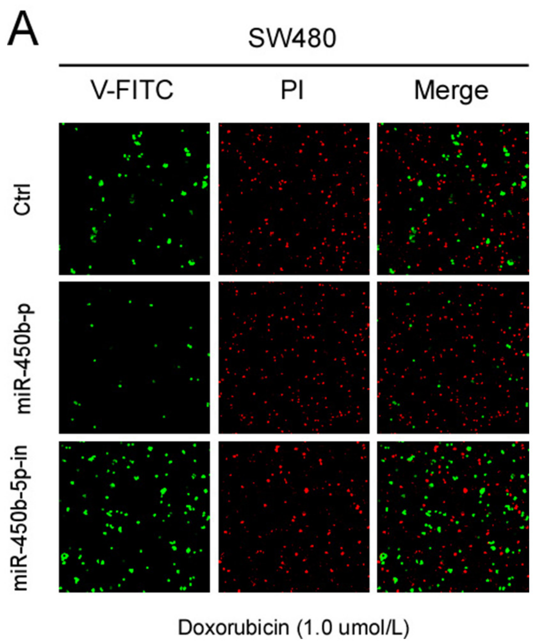

Supplementary Figure S6: miR-450b-5p inhibits apoptosis of CRC cells. Annexin V-FITC and PI staining performed on indicated cells treated with 1.0μM doxorubicin. Annexin-positive/PI-negative cells were considered apoptisic cells.

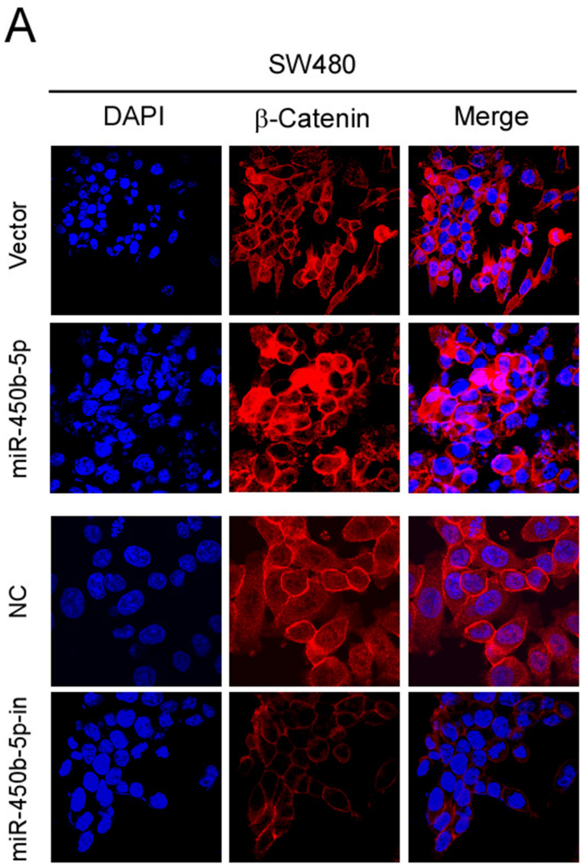

Supplementary Figure S7: miR-450b-5p promotes beita-catenin into the nucleus in CRC cells. Confocal laser scanning technique performed on indicated cells to observe subcellular localization of beita-catenin in CRC cells transfected with miR-450b-5p and miR-450b-5p-inhibitor.

Supplementary Table S1: Correlation between clinicopathological features and miR-450b-5p expression in 170 CRC tissues

Characteristics	miR-450b-5p expression		P value
	Low	High	
Age			
≤mean (66)	47	40	0.284
>mean (66)	38	45	
Gender			
Male	40	41	0.878
Female	45	44	
T classification			
1-2	44	14	<0.001
3-4	41	71	
N classification			
0	70	7	<0.001
1-2	15	78	
M classification			
M0	79	49	<0.001
M1	6	36	

Supplementary Table S2: Spearman correlation analysis between Relative miR-450b-5p expression and Clinicopathologic Features

Variables	Relative miR-450b-5p expression	
	Spearman correlation	*P*-value
T classification	0.372	<0.001
N classification	0.744	<0.001
M classification	0.409	<0.001

Supplementary Table S3: Univariate and Multivariate analyses of various prognosis parameters in 170 CRC patients using Cox Regression model

variable	Category	No. Patients	Univariate analysis		Multivariate analysis		
			P	S.E	*P*	Relative Risk	95%CI
miR-450b-5p	low	85	0.000	0.275	0.008	2.649	1.294-5.420
	high	85					
T classification	1-2	58	0.000	0.144	0.002	1.802	1.249-2.598
	3-4	112					
N classification	0	77	0.000	0.146	0.976	1.016	0.473-2.183
	1-2	93					
M classification	0	128	0.001	0.875	0.791	0.875	0.325-2.356
	1	42					

SE: standard error; RR: relative risk; CI: confidence interval

Supplementary Table S4: Primer sequences used for Real-time PCR (5' to 3')

Gene	Forward primer	Reverse primer
SIAH1	TGCTGTTGACTGGGTGAT	TTGCTTGCGTGTTCCTAT
SFRP2	CCAGCCCGACTTCTCCTA	GCTCCAGCACCTCCTTCA
p27Kip1	CCGGTGGACCACGAAGAGT	GCTCGCCTCTTCCATGTCTC
p21Cip1	ACATCGCCAAGGAAAAACGC	GTCTGTTTCGGTACTGTCATCC
cyclinD1	CCGTCCATGCGGAAGATC	ATGGCCAGCGGGAAGAC
GAPDH	GACTCATGACCACAGTCCATGC	AGAGGCAGGGATGATGTTCTG

Supplementary Table S5: Primer sequences used for amplification and plasmid construction (5' to 3')

Gene	Forward primer	Reverse primer
miR-450b-5p	CTCAACTGGTGTCGTGGAGTCGG CAATTCAGTTGAGTATTCAGG	ACACTCCAGCTGGGTTTTG CAATATGTTCC
SIAH1-3'UTR-WT	.ATCGACGCGTCGTGTTC AATATTTTCTTTTCCCCC	GATAGCTAGCTGCCAGATATTC TTTTAATGAG
SFRP2-3'UTR-WT	ATAGACGCGTCTG AGGTTGTCATAACAT	GCTAGCTAGCATAGGTAAA ACAGGATGT

Supplementary Table S6: Primer sequences used for ChIP analysis of the binding between AP-1 and miR-450b-5p promoter. (5' to 3')

	Forward primer	Reverse primer
Primer 1	GTGGGAGGATGAGTTGTG	TGTTTTCCAGTTGTTTGC
Primer 2	AAAACACTTTGCTATAAGC	TAATCCTTTGTTGTTGGT
Primer 3	ACAAATAAAATACAGCGTAG	TCATACAGAATGCTCCCT
Primer 4	TACAGATAAAAGGCTAGG	CAAAATGTCCCCAATACA
Primer 5	AAGGTGACCAAAGAAAGA	TTGATTGCCTATAGTGAC
Primer 6	CCAAAAGTCTAGAGGTGG	AGATAGCCGCTGTTAGAT
Primer 7	GATGAAAAGATGGAGGGA	ACTATGGATGCAAAATGA
